# Preparatory activity for purposeful arm movements in the dorsomedial parietal area V6A: Beyond the online guidance of movement

**DOI:** 10.1038/s41598-018-25117-0

**Published:** 2018-05-02

**Authors:** Elisa Santandrea, Rossella Breveglieri, Annalisa Bosco, Claudio Galletti, Patrizia Fattori

**Affiliations:** 1 Department of Biomedical and Neuromotor Sciences, University of Bologna, Bologna, Italy; 2 Department of Neuroscience, Biomedicine and Movement Sciences, University of Verona, Verona, Italy

## Abstract

Over the years, electrophysiological recordings in macaque monkeys performing visuomotor tasks brought about accumulating evidence for the expression of neuronal properties (e.g., selectivity in the visuospatial and somatosensory domains, encoding of visual affordances and motor cues) in the posterior parietal area V6A that characterize it as an ideal neural substrate for online control of prehension. Interestingly, neuroimaging studies suggested a role of putative human V6A also in action preparation; moreover, pre-movement population activity in monkey V6A has been recently shown to convey grip-related information for upcoming grasping. Here we directly test whether macaque V6A neurons encode preparatory signals that effectively differentiate between dissimilar actions before movement. We recorded the activity of single V6A neurons during execution of two visuomotor tasks requiring either reach-to-press or reach-to-grasp movements in different background conditions, and described the nature and temporal dynamics of V6A activity preceding movement execution. We found striking consistency in neural discharges measured during pre-movement and movement epochs, suggesting that the former is a preparatory activity exquisitely linked to the subsequent execution of particular motor actions. These findings strongly support a role of V6A beyond the online guidance of movement, with preparatory activity implementing suitable motor programs that subsequently support action execution.

## Introduction

Area V6A of the macaque monkey, which lays in the caudalmost part of the superior parietal lobule^1,2^, is a critical node within the dorsomedial visual stream. It is known to integrate visual and somatosensory information at the service of online guidance of prehensile motor actions^3,4^. Thanks to a systematic accumulation of evidence in studies where single-unit recordings were performed during the execution of appropriate visuomotor tasks, V6A neurons have been demonstrated to express several properties which are perfectly suitable for encoding and orchestrating prehension^4^. In particular, in addition to expressing selectivity in the visuospatial^5–9^ and somatosensory^10,11^ domains, V6A neurons were shown to encode visual affordances^12^ and motor cues that allow monitoring direction of arm reaching and shaping of hand/grip configuration for grasping movements^4,13–19^. Critically, while the dorsomedial visual pathway has long been known to support encoding of reaching movements^8,14,20–31^, a role of this pathway in encoding grasping movements has been more recently acknowledged. Specifically, after the first description of grasp-related activity in area V6A^3,13^, neurons in this area have been shown to selectively encode wrist orientation^15^ and grip type^16,17^.

Beyond its established functional role in the online guidance of arm/hand actions^4,32^, V6A has been proposed to be also involved in motor preparation. Recent imaging studies with healthy human volunteers demonstrated that the putative human homologue of area V6A^33–35^, which is likely located in the anterior part of the superior parieto-occipital cortex (aSPOC)^36–38^, shows enhanced visual activation to objects presented within the peripersonal “graspable” space, even when the potential action is not actually executed^39^. These data demonstrate a role of this area in encoding the behavioral relevance of objects for hand actions, well in accordance with the recent finding of an involvement of V6A neurons in encoding visual affordances^12^. Other recent human fMRI studies have further suggested that aSPOC might have a critical role in the transformation of information relative to visual affordances into a specific motor program^38,40^. Interestingly, decoding of pre-movement activity of aSPOC with fMRI pattern analysis allowed reliable classification of specific actions that were subsequently performed, with a clear distinction between reaching and grasping movements^40^. In essence, activity measured in aSPOC before movement execution seems to selectively encode specific motor programs that require planning of arm direction, hand preshaping and grip selection^38,40^, in other words movement preparation. Along these lines, recent studies in the macaque monkey have demonstrated that information for grasping, notably differentiating grip configurations, is reliably decoded from population neural activity acquired from macaque area V6A in a pre-movement phase (as well as during other task epochs) of a grasping task^41,42^.

Neural activity in the macaque V6A has been previously investigated in the pre-movement phase in the context of instructed-delay reaching^43^ and grasping^15^ tasks, separately. In particular, in a previous paper, we demonstrated that activity in a pre-movement phase could be modulated by the specific wrist orientation required in the subsequent grasping action, based on variations in the orientation of the to-be-grasped handle^15^. However, single-unit electrophysiological evidence in the macaque area V6A attesting to differential preparatory activity for actions aimed at pressing an object (not requiring any specific hand-object interaction, but simply aiming at the specific spatial location of the object) or at grasping an object (requiring accurate hand shaping to allow object acquisition in the hand) is still lacking. A clear demonstration of such a differential preparatory activity in single V6A neurons would help establish that the functional role of V6A, going beyond the online guidance of movement, also involves action preparation and actively encodes the subsequent act of prehension.

Within this framework, here we want to directly test whether V6A neurons encode preparatory signals that are specifically related to the action to be subsequently performed, thus effectively differentiating types of action in advance of movement execution. Specifically, we recorded activity of single V6A neurons during the execution of two instructed-delay visuomotor tasks in which the monkeys were instructed to perform either arm/hand movements aimed at reaching and pressing a visual target (*Reach-to-Press task*), or arm/hand movements aimed at reaching and grasping a handle (*Reach-to-Grasp task*). As we aimed to establish whether any modulations arising in advance of movement execution were specifically related to the action to be performed, notably in relation to the required hand shaping adjustments, the two tasks were constructed in a way to involve the same temporal and spatial structure. Arm/hand movements were performed in different background conditions, namely in the dark and in the light, to control for any influence of visual information and, more importantly, to look for a consistent task-related modulation of activity across different visual contexts, key to the demonstration of an action-specific signal in a pre-movement phase. We found that V6A activity preceding the onset of arm movement was exquisitely linked to the subsequent execution of specific motor actions, notably reach-to-press vs. reach-to-grasp movements, thus revealing to be a preparatory activity. In line with previous demonstrations of a sensitivity of V6A neurons to visual feedback^7,18,19^, a specific task-related activity in a pre-movement phase – the focus of the present paper – was measured either alone or in combination with modulations of neuronal discharge by the presence/absence of visual information.

## Results

**Figure 1:**
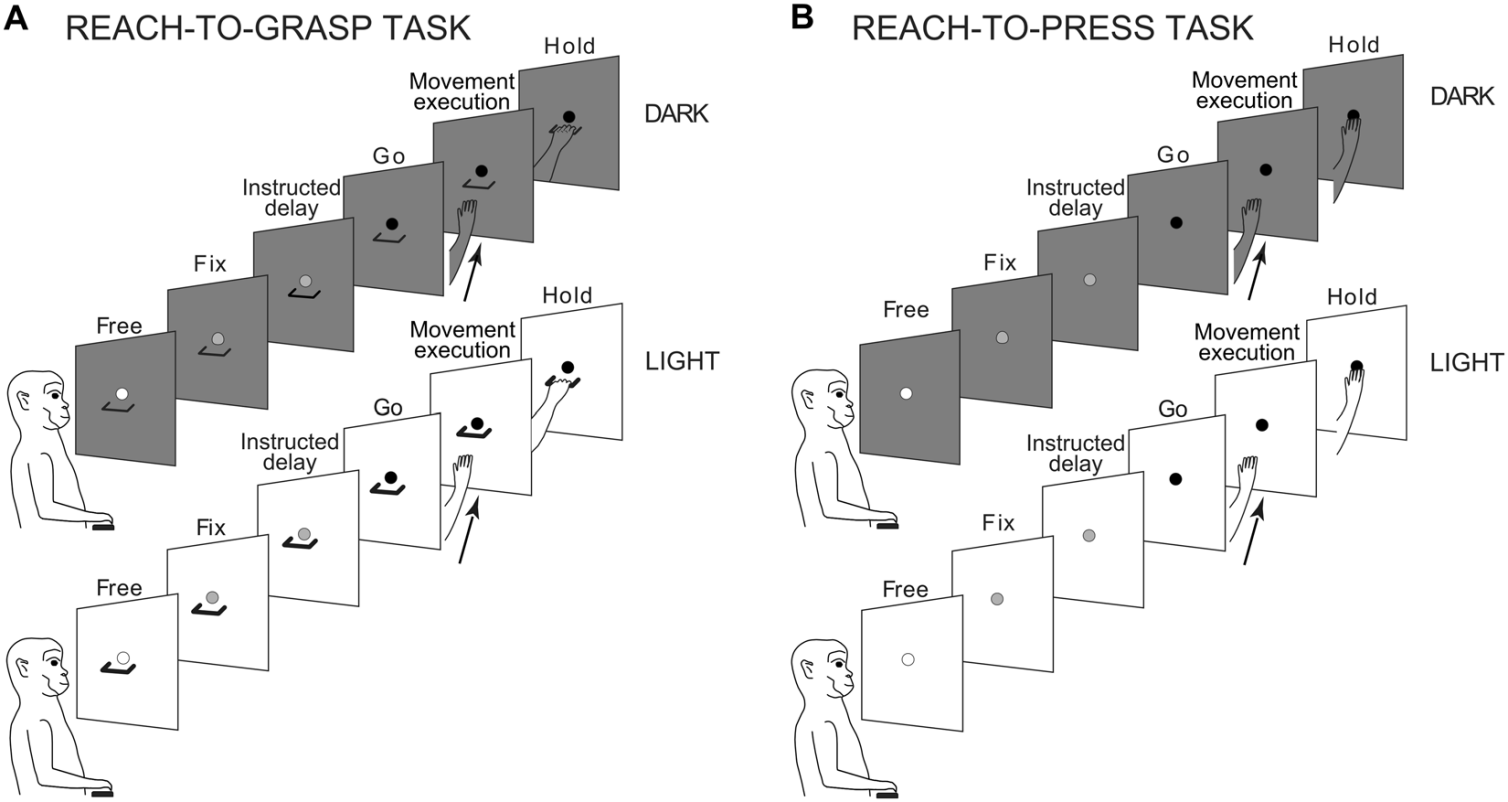
Behavioral tasks. (**A,B**) The figure represents the unfolding of events occurring during an example trial of the Reach-to-Grasp task (**A**) and of the Reach-to-Press task (**B**), either executed in the dark (upper panels) or in a light background (lower panels). For both tasks, each panel in the sequence corresponds to different events in the trial, as referred to by a specific label (see Methods for a detailed description of the tasks). In brief, each trial sequence began when the monkey pressed a home-button placed outside its field of view; after button press, the animal awaited instructions while still being free to move the eyes (FREE). After 0.5–1 s, a green fixation LED lit up and the monkey was required to fixate it (FIX) and to maintain fixation thereafter. After a variable delay period (1–2.5 s; INSTRUCTED-DELAY), the LED color changed from green to red (GO); this GO signal instructed the monkey to perform either a reach-to-press or a reach-to-grasp movement (MOVEMENT EXECUTION). The monkey was then asked to maintain its arm/hand in targeted position (HOLD) until the red LED switched off again, instructing the monkey to press again the home-button in order to obtain a liquid reward for correct performance and to then start a new trial sequence. Note that the last part of the instructed-delay phase, defined as the DELAY epoch, and an epoch comprising movement execution, defined as MOV epoch, were considered as the critical epochs of interest in the analyses, as detailed in the text.

We trained two macaque monkeys to perform a Reach-to-Grasp task (requiring to reach and grasp a handle) and a Reach-to-Press task (requiring to reach and press a button), in separate blocks of trials (Fig. 1). As illustrated in details in the Methods, the two instructed-delay visuomotor tasks were identical in terms of spatial and temporal sequence and procedure, but required different hand actions. Such an experimental design was chosen to ensure the possibility to exclude that any selectivity for a given task in a pre-movement phase of the trial was associated to factors other than the type of impending arm/hand movement. First, the identical spatio-temporal structure of the two tasks was aimed to exclude any influence of factors such as arm direction or target location (which are known to have a strong impact on neuronal activity in V6A^14,43–47^) in determining any diverging pattern of activity. In fact, the two tasks (Reach-to-Press and Reach-to-Grasp) can be considered identical in terms of arm direction and target location (i.e., they share a highly similar transport component, corresponding to the reaching phase of the movement), while they differ in terms of the required hand/grip configuration (i.e., they are strongly divergent in terms of the distal component involved, thus requiring differential encoding of hand shaping). Second, we avoided possible confounding effects related to eye position (another factor which is known to affect ongoing neural activity in area V6A^43,48–50^) by requiring stable fixation along the whole duration of the trials, therefore keeping the gaze fixed in a consistent spatial location across tasks. Third, the Reach-to-Grasp task was designed as to evoke grasping actions that required keeping the wrist pronated, as it is the case in the Reach-to-Press task, in order to exclude that modulations of V6A neural activity might simply reflect coding of wrist orientation (yet another factor which strongly affects neural activity in V6A in both pre-movement and movement phases of a visuomotor task^15^).

After completion of the training phase, recording sessions were carried out while the animals performed the learned visuomotor tasks in different background conditions (Fig. 1), in separate blocks of trials (see Methods). Complete recordings in both tasks and in both background conditions were obtained for 91 neurons, isolated within the limits of area V6A. Out of these 91 cells, 85 were selected for in-depth quantitative analyses after a preliminary screening aimed at assessing the fulfillment of inclusion criteria. The selected database comprised recordings with good signal quality and stability, and a reliable number of repetitions (typically 10–15) for the whole set of 4 experimental conditions, i.e., two experimental tasks, each performed in two background conditions (see Methods).

**Figure 2:**
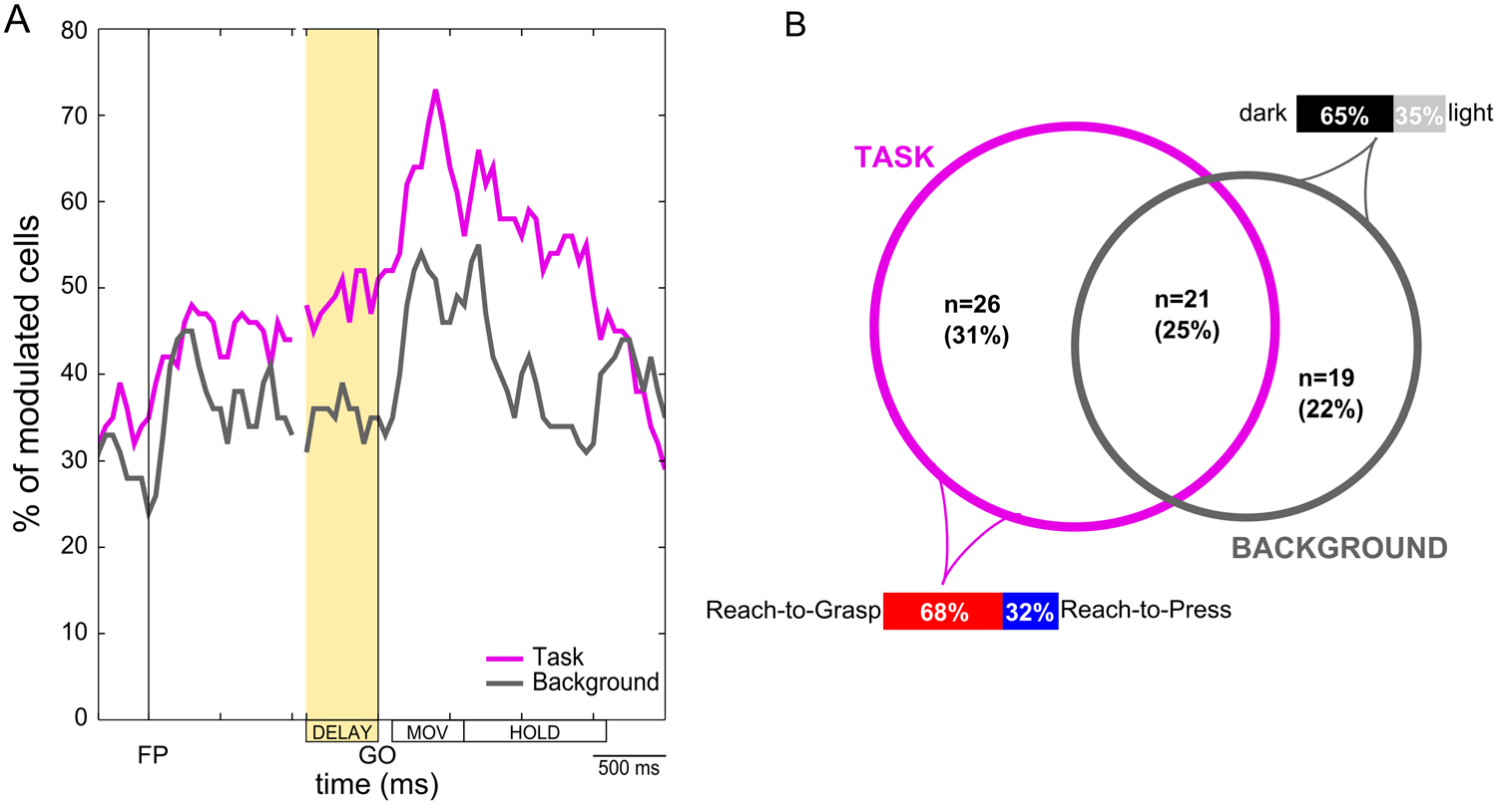
General assessment of modulations in the population of V6A neurons. (**A**) Percentage of cells in the population with task preference (magenta) and background sensitivity (dark gray) in sliding windows (width: 200 ms, shift: 50 ms) covering the whole duration of the trial (sliding window ANOVA, p < 0.05). (**B**) Schematics of task-related and background-related modulations measured during the DELAY epoch. The graph reports the number and percentage of cells showing a main effect of Task (magenta circle), a main effect of Background (dark gray circle) or both effects (intersection) (repeated-measures ANOVA, p < 0.05). Colored bands represent the general incidence of task preference for reach-to-grasp (68% considering all task-selective cells; red) vs. reach-to-press (32% considering all task-selective cells; blue) movements (bottom left), and the incidence of background preference for dark (65% considering all background-selective cells; black) or light (35% considering all background-selective cells; light gray) background (up right), respectively, within the whole population of modulated neurons.

We first explored the overall impact of task-related and background-related modulations in our population of neurons by applying a repeated-measure ANOVA, with the within-subject factors *Task* (Reach-to-Press vs. Reach-to-Grasp) and *Background* (dark vs. light), to average spiking activity measured in progressively shifted 200 ms windows^51^ (see Methods), covering the whole duration of the trial. As shown in Fig. 2A (in magenta), the percentage of cells with reliable Task preference grew steadily during the first part of the trial, soon after the animal acquired central fixation, reaching a consistent fraction (~50%) during the instructed-delay phase of the task (see Methods), and notably during the DELAY epoch (i.e., the last 500 ms before the GO signal instructing movement execution; yellow area in the graph). The incidence of Task preference then peaked after the GO signal, during actual movement execution (MOV epoch, i.e. from 200 ms before movement onset to movement end; see Methods), when the majority (60–70%) of the cells in the population showed a significant main effect of Task (p < 0.05). Thereafter, the occurrence of Task effects in the population slowly decreased (HOLD epoch, i.e. when the monkey held the hand on the target; see Methods). As far as sensitivity to visual background is concerned, we found a lower incidence of reliable Background preference, but a similar time course of the effect (Fig. 2A, dark gray curve). Specifically, the percentage of cells with a significant main effect of Background quickly rose to ~40% around the time when the animal acquired central fixation after the presentation of the fixation LED (FP), then settling at an incidence of ~35% for the entire duration of the instructed-delay phase of the task. Subsequently, a peak in the incidence of background preference (~50% of cells) was observed during the MOV epoch, and then the incidence of cell modulation decreased during the HOLD epoch. Overall, the activity of V6A neurons in our population was modulated by both the experimental task, hence the impending reach-to-press or reach-to-grasp movement, and the background condition in which the task was executed. However, task-related modulations were stronger than background-related ones in all parts of the trial.

To directly assess whether the spiking activity which preceded the GO signal in the given task was modulated by the type of action to be subsequently performed and/or by the background condition, we then focused on single-cell firing rate in the DELAY epoch (from −500 to 0 ms before the GO signal). In details, we performed a repeated-measure ANOVA, as previously described, on the DELAY activity of single cells and evaluated the main effects (and interactions) of the considered factors (Fig. 2B). We found that 26/85 (31%) cells (of which 9 also showed a reliable interaction) were significantly modulated by the Task (for now on *Task cells*), 19/85 (22%) cells (of which 4 also showed a reliable interaction) by the Background (for now on *Background cells*), and 21/85 (25%) cells (of which 12 also showed a reliable interaction) by both (for now on *Task/Background cells*). Note that in most cases, for all the above mentioned groups of cells, when an interaction was present in addition to main effects, task preference was more pronounced in one vs. the other background condition (with either the larger effect being measured in the dark or in the light condition), although fully coherent across them. For the remaining 19/85 cells (22%), none of the considered factors yielded a significant independent modulation. However, 6 of these cells (7% in the whole population) showed a pure interaction between the two factors (in the absence of any main effects), which in most cases corresponded to a (slight) inversion in task preference during the delay depending on the background condition, most likely reflecting lack of a fundamental role in encoding task-related signals by the given cell. Given the small fraction of neurons with a pure interaction, and the fragility of the observed pattern, we did not consider this subgroup of cells of any further interest for our characterization of pre-movement activity in V6A.

Further analyses were conducted separately for the first three groups of cells defined above, i.e. those with a main effect of Task, a main effect of Background or both independent modulations. For *Background cells*, results were in full agreement with previous data reported from our laboratory^7,18,19^, showing that the majority of them (11/19, 58%) had a preference for the absence of visual feedback, i.e. they were more active in the dark. Therefore, in what follows, we will not describe in details the pattern of discharge of this type of cells, focusing instead on *Task* and *Task/Background* cells. Note that, in the context of the present paper, collecting data in two background conditions was specifically meant as an experimental stratagem for a well-controlled investigation of the functional role of task-related activity in a pre-movement phase. In particular, a consistent task-related modulation of activity across background conditions (main effect of Task) can be rather convincingly interpreted as the reflection of an action-specific signal, whereas the same task preference assessed only in one visual context might be subject to different interpretations.

### Task cells: selective encoding of the forthcoming arm/hand action

To gain a deeper understanding of the nature and functional meaning of pre-movement task-related modulations, we first concentrated on *Task cells*, i.e. on the group of V6A cells for which activity during the DELAY epoch resulted to be significantly modulated by the factor Task in isolation (Fig. 2B).

**Figure 3:**
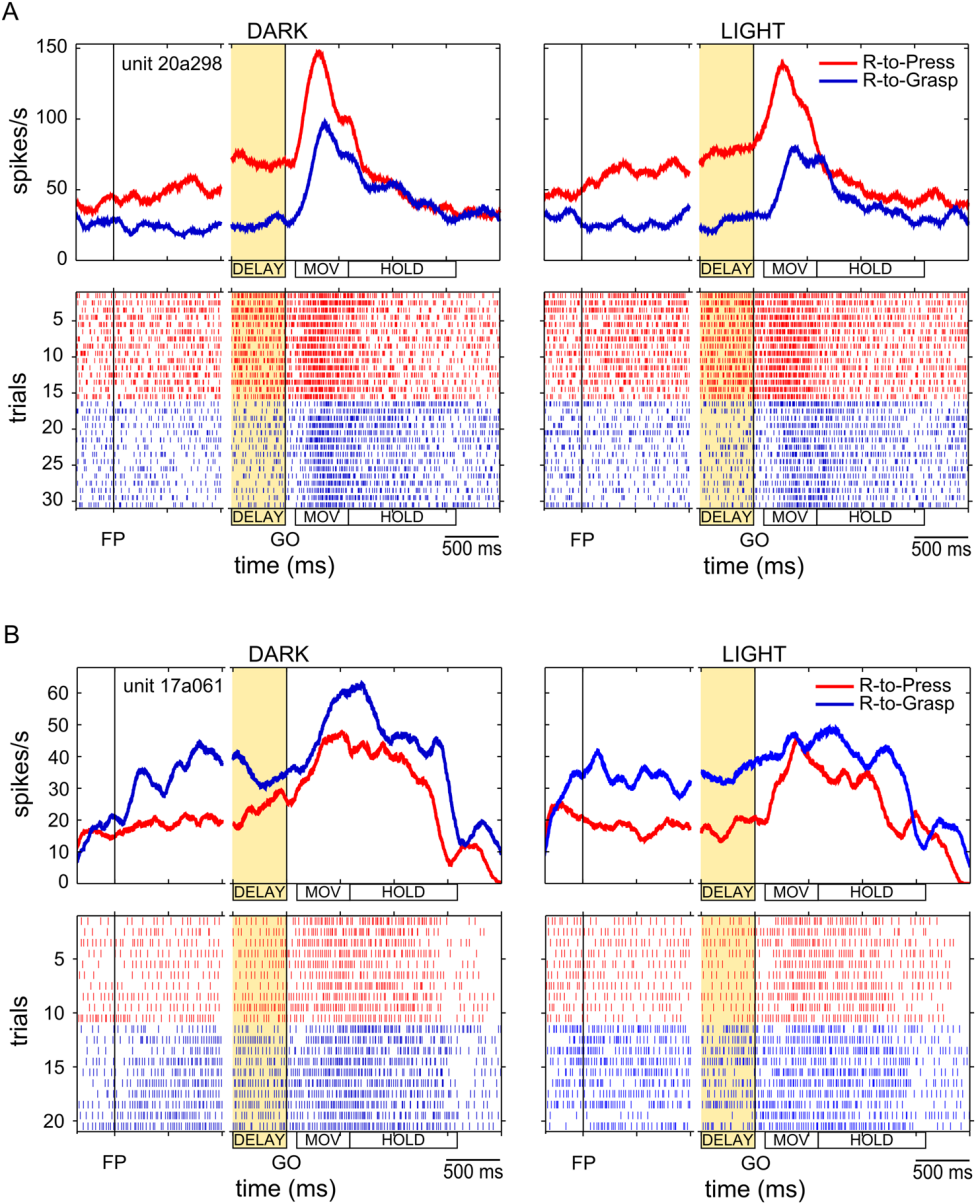
Examples of single neurons classified as *Task cells*. (**A**) Example of *Task cell* showing a preference for reach-to-press movements. Activity is shown separately for the Reach-to-Grasp Task (in blue; “R-to-Grasp”) and for the Reach-to-Press task (in red, “R-to-Press”), either when executed in a dark (left panels) or in a light background (right panels). Both SDFs (upper panels) and raster plots of impulse activity (lower panels) are shown for each experimental condition. Neural data from single trials were realigned twice, first to the onset of the fixation LED (FP, left vertical line) and then to the GO signal (GO, right vertical line). The yellow area on the graphs represents the time epoch of major interest for the purpose of the present study, i.e. the DELAY epoch, within which a significant modulation of activity by the factor Task was assessed for the depicted cell. Scale: vertical bar on SDFs, 153 spikes/s. (**B**) Example of *Task cell* showing a preference for reach-to-grasp movements. All conventions as in panel A. Scale: vertical bar on SDFs, 68 spikes/s.

Figure 3 depicts two representative, single-cell examples from this subpopulation. The neuron depicted in Fig. 3A showed higher discharge during the Reach-to-Press (vs. Reach-to-Grasp) task, both in the dark (on the left) and in the light (on the right). The task-related modulation was particularly evident during the DELAY epoch (yellow area), the critical time window of our interest, and during movement execution (MOV epoch). Instead, the neuron depicted in Fig. 3B showed higher discharge during the Reach-to-Grasp (vs. Reach-to-Press) task, again both in the dark (on the left) and in the light (on the right). The task-related activity modulation was well evident during the DELAY epoch (yellow area) and was maintained after the GO signal, throughout the execution of the arm/hand movement, both in dark and light background conditions.

Among *Task cells*, a preference for the Reach-to-Grasp task was more frequent than for the Reach-to-Press task (69% vs. 31% of *Task cells*). The degree of task selectivity during DELAY, as assessed by the Task Preference Index (TPI; see Methods), was comparable in the two subpopulations (0.34 ± 0.05 SEM for neurons preferring Reach-to-Press; −0.31 ± 0.03 SEM for neurons preferring Reach-to-Grasp; p = 0.60, two-tailed unpaired t-test comparing absolute TPI values).

**Figure 4:**
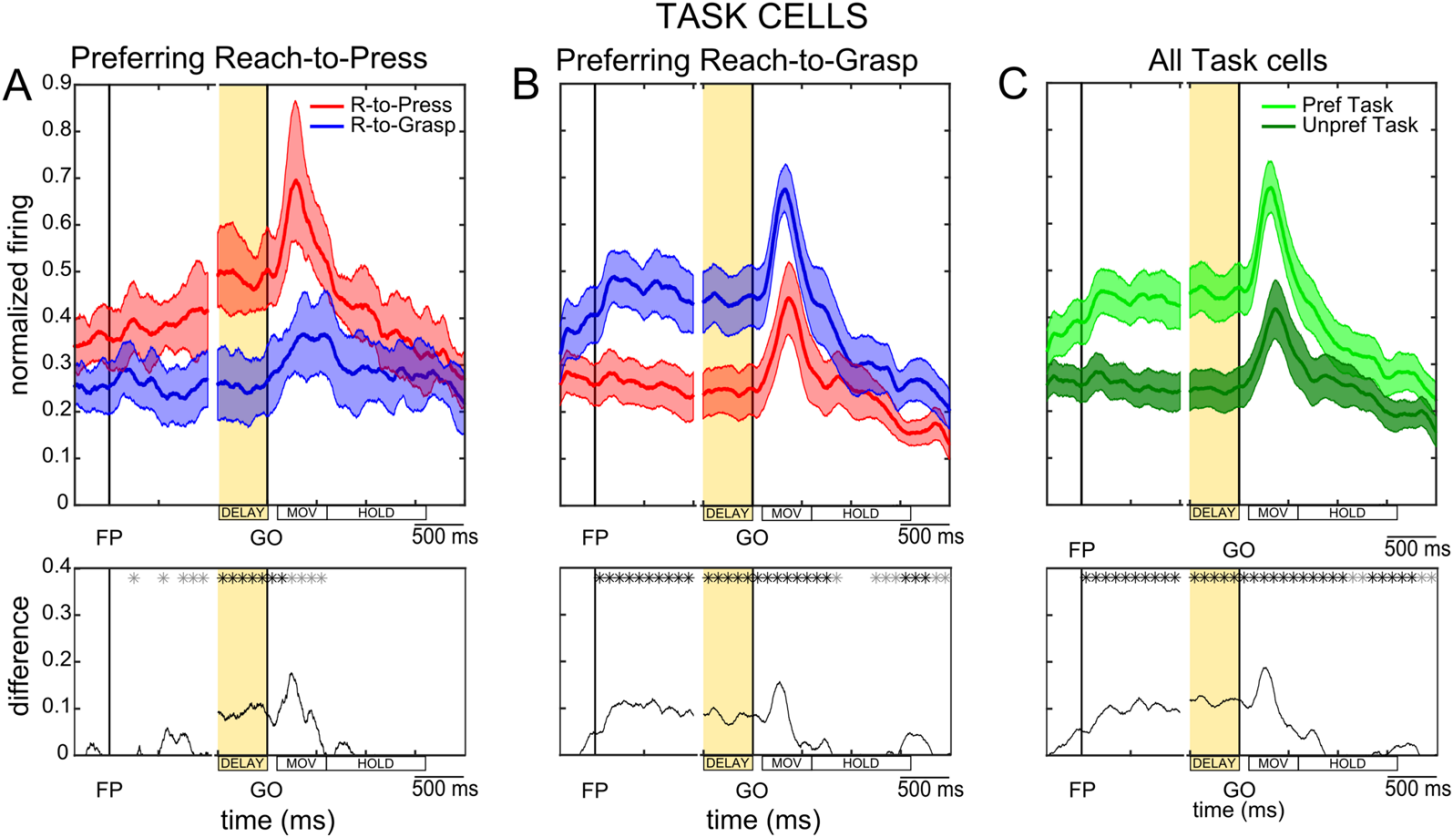
Task-related modulations during the DELAY epoch. (**A**) Average population activity for *Task cells* showing a preference for reach-to-press movements. In the upper panel, population SDFs are shown separately for the Reach-to-Grasp (in blue) and the Reach-to-Press (in red) tasks, averaged across background conditions. Alignment of neural data was identical to what described for Fig. 3. Shaded red and blue areas indicate 95% confidence intervals (CI) computed using a subsampling procedure (see Methods). In the lower panel, the thin black line represents positive values (>0) of the difference between the lower CI limit of the curve identifying the preferred task and the upper CI limit of the curve identifying the non-preferred task. Asterisks reported on the same graph represent instead the results of two-tailed paired Wilcoxon tests comparing experimental conditions, performed on the activity measured in consecutive 100 ms time windows along the whole duration of the trial, beginning after the presentation of the fixation LED (epoch FIX); * in gray = p < 0.05; * in black = p < 0.01. (**B**) Average population activity for *Task cells* showing a preference for reach-to-grasp movements. All conventions as in panel A. (**C**) Population SDFs represent the temporal unfolding of task preference across the whole population of *Task cells*, as assessed by averaging spiking activity during the preferred (green) vs. unpreferred task (dark green), again across background conditions. All conventions as in panel A.

Normalized population SDFs were separately constructed for the *Task cells* with a preference for Reach-to-Press (Fig. 4A) and for those with a preference for Reach-to-Grasp (Fig. 4B), averaging the activity across the two visual background conditions. To assess consistency of the results across cells in each population, we applied a subsampling method for bootstrapping data (see Methods) and estimated the resulting 95% confidence intervals for each curve (shaded areas in the figure). As reported in Fig. 4A and B, by this approach we confirmed that a substantial and reliable difference in the activity recorded during the two tasks was present during the instructed-delay phase of the task, as well as during movement execution (see black lines in the lower panels of each figure; for details, see Methods). A small but significant difference in activity remained also after action completion, i.e. at the very beginning of the HOLD epoch (Fig. 4A,B). It is important to note that, in this later phase of the trial, while the animal held the object by the whole hand in the Reach-to-Grasp task (the hand was wrapping around the object), it pushed the button with only the digit tip in the Reach-to-Press task; critically, the difference in discharge between the two conditions is null for most of the duration of the HOLD (when the movement has ceased) and the firing rates measured across tasks overlap in most cells. Interestingly, for cells preferring reach-to-grasp actions (Fig. 4B), the difference in discharge between the two tasks emerges again at the end of the HOLD epoch, when the fingers are extended to release the object in the grasping task (while simply detaching from the button in the pressing task). This observation denotes that the firing rate in V6A does not parallel the somatosensory inputs derived from the grasping vs. pressing action, although somatosensory stimulations are effective in V6A neurons^10,11^; however, the analysis of this signal is outside the scope of the present research, which is focused on the activity during pre-movement phase and its putative relationship with the activity during the actual movement phase.

To further test the reliability of modulations reported in Fig. 4A and B, two-tailed paired Wilcoxon tests were applied to compare population firing measured in the two tasks in subsequent 100 ms windows along the entire duration of the trial (starting from the onset of the fixation LED, FP), which led to fully convergent results (asterisks in the lower panels of Fig. 4A and B indicate the significance of the described statistical test; * in gray = p < 0.05; * in black = p < 0.01).

Interestingly, Fig. 4A and B show that task selectivity (a reliable difference in cell activity measured across tasks) emerged soon after the onset of the fixation LED (FP, left vertical line; see epoch FIX in Fig. 1). In cells preferring Reach-to-Grasp (Fig. 4B), task selectivity emerged earlier and was stronger in the initial phase of the trial with respect to what observed in cells preferring Reach-to-Press (Fig. 4A). This precocious selectivity is likely related to the responsiveness of V6A neurons to visual affordances of objects to be grasped^12,17^, and/or to a stronger engagement of working memory in the case of the Reach-to-Grasp task (see Discussion).

Task selectivity seems to emerge even before the onset of fixation (especially for cells preferring Reach-to-Grasp; Fig. 4B); this is likely related to the fact that tasks were performed in blocks, thus the animals knew in advance whether a reach-to-press or a reach-to-grasp movement was required in the given trial, which allowed precocious retrieval of movement-related information from memory and advanced movement planning.

Population SDFs depicted in Fig. 4C represent the temporal unfolding of task preference across the whole population of *Task cells* (either preferring Reach-to-Press or Reach-to-Grasp), as assessed by averaging spiking activity during the preferred vs. unpreferred task, again across visual background conditions. As assessed both by the subsampling method (black line in the lower panel of the figure; see Methods) and non-parametric statistical testing (asterisks in the lower panel of the figure; see Methods), the activity clearly diverged during the instructed-delay epoch, as well as during the subsequent execution of the required movement, and remained significantly different during the early (and late; see above) phase of the HOLD epoch (following the completion of the arm/hand action). Given the prevalence of neurons preferring Reach-to-Grasp in the population, the overall activity measured in the whole population of *Task cells* (Fig. 4C) shows a time course similar to that depicted in Fig. 4B, with a precocious emergence of task preference.

**Figure 5:**
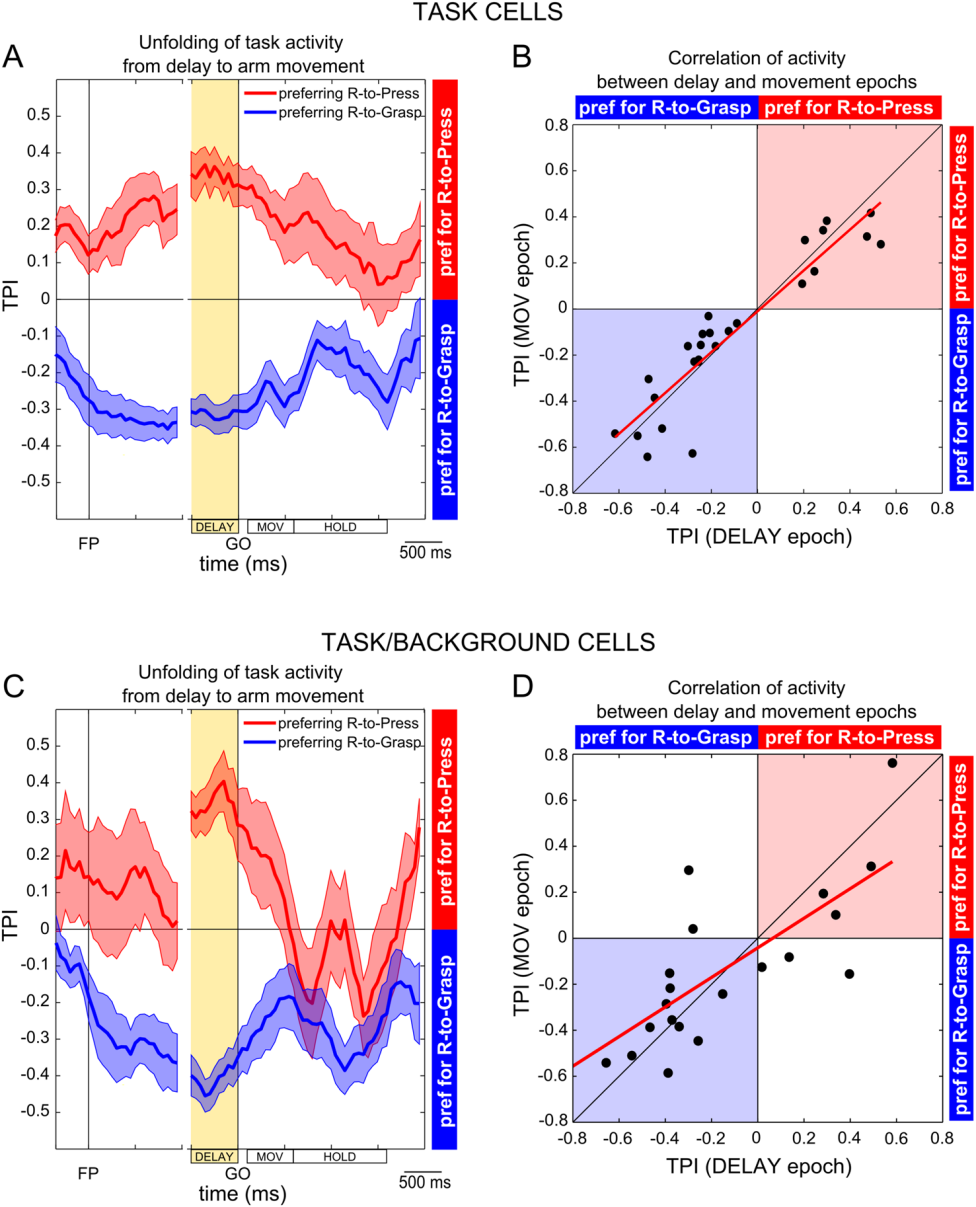
Temporal dynamics of task-related activity and consistent task-preference during different time epochs. (**A**) Sliding TPI (window: 200 ms; shift: 50 ms) along the entire duration of the trial was computed on average across *Task cells* showing a preference for reach-to-press movements during DELAY (in red) and across *Task cells* showing a preference for reach-to-grasp movements during the same epoch (in blue). Note that TPI values > 0 indicate a preference for reach-to-press movements, while TPI values < 0 indicate a preference for reach-to-grasp movements (as indicated by lateral colored bands); values approximating 0 indicate no difference in neural activity across the two tasks (see Methods). (**B**) Scatter-plot comparing single-cell TPI values computed on average neuronal activity during the DELAY epoch and during movement execution (epoch MOV; see Methods and Fig. 1) in the population of *Task cells* and best-fitted linear function (TPI_MOV_ = −0.011 + 0.885 * TPI_DELAY_; red line; see Results). Note that data points laying in the upper right quadrant represent neurons with consistent preference for Reach-to-Press during the DELAY and MOV epochs; analogously, data points laying in the lower left quadrant represent neurons with consistent preference for Reach-to-Grasp during the two epochs. (**C**) Sliding TPI (window: 200 ms; shift: 50 ms) along the entire duration of the trial was computed on average across *Task/Background cells* showing a preference for reach-to-press movements during DELAY (in red) and across *Task/Background cells* showing a preference for reach-to-grasp movements during the same epoch (in blue). All conventions as in panel A. (**D**), Scatter-plot comparing single-cell TPI values computed on average neuronal activity during the DELAY epoch and during movement execution (epoch MOV; see Methods) in the population of *Task/Background cells* and best-fitted linear function (TPI_MOV  _= −0.042 + 0.643* TPI_DELAY_; red line; see Results). All conventions as in panel B.

To gain a deeper understanding of the precise unfolding of task-related activity, we analyzed how the task-preference changed along the trial by computing a Task Preference Index (TPI; see Methods) in sliding 200 ms time windows, progressively shifted by 50 ms. Results from this analysis are reported in Fig. 5A, separately for the cells that showed a preference for the Reach-to-Press task (red line; n = 8) and for those preferring the Reach-to-Grasp task (blue line; n = 18). The maximal expression of task preference (maximal absolute value of TPI; see Methods) was found during DELAY, and high value of task preference was maintained during the execution of arm/hand movement, for both subgroups of cells.

Our main interest here was to gain understanding of the functional role of the DELAY activity. Given that one likely possibility is that it represents encoding of preparatory activity for upcoming motor actions, we looked for evidence of a consistency at the single cell level between the task-related activity expressed during the DELAY epoch and that expressed subsequently, during movement execution. To this aim, we plotted the single-cell TPI measured during the DELAY epoch against the TPI measured during the MOV epoch for the whole population of *Task cells* (n = 26; Fig. 5B). The linear regression analysis of the data showed a strong and highly significant relationship between TPI measures across the two epochs, as described by a slope value of 0.88 and a R^2^ of 0.85 (F = 140.98, p < 0.0001; Fig. 5B), with all data points laying either in the upper right quadrant (consistent preference for Reach-to-Press) or in the lower left quadrant (consistent preference for Reach-to-Grasp). The striking consistency of task-related activity during the two epochs at the single cell-level strengthens the idea that the modulation we observed in *Task cells* during DELAY can be qualified as a preparatory activity related to the planning of the specific arm/hand action to be subsequently executed.

### Combined selectivity for task and background conditions

We called *Task/Background cells* the neurons showing both task-related and background-related activity during the DELAY epoch. Among those cells, 14/21 (67%) showed a preference for the Reach-to-Grasp task (10 cells were more active when the task was executed in the dark and 4 in the light). The remaining 7 neurons (33%) showed a preference for the Reach-to-Press task (5 cells were more active when the task was executed in the dark and 2 in the light).

**Figure 6:**
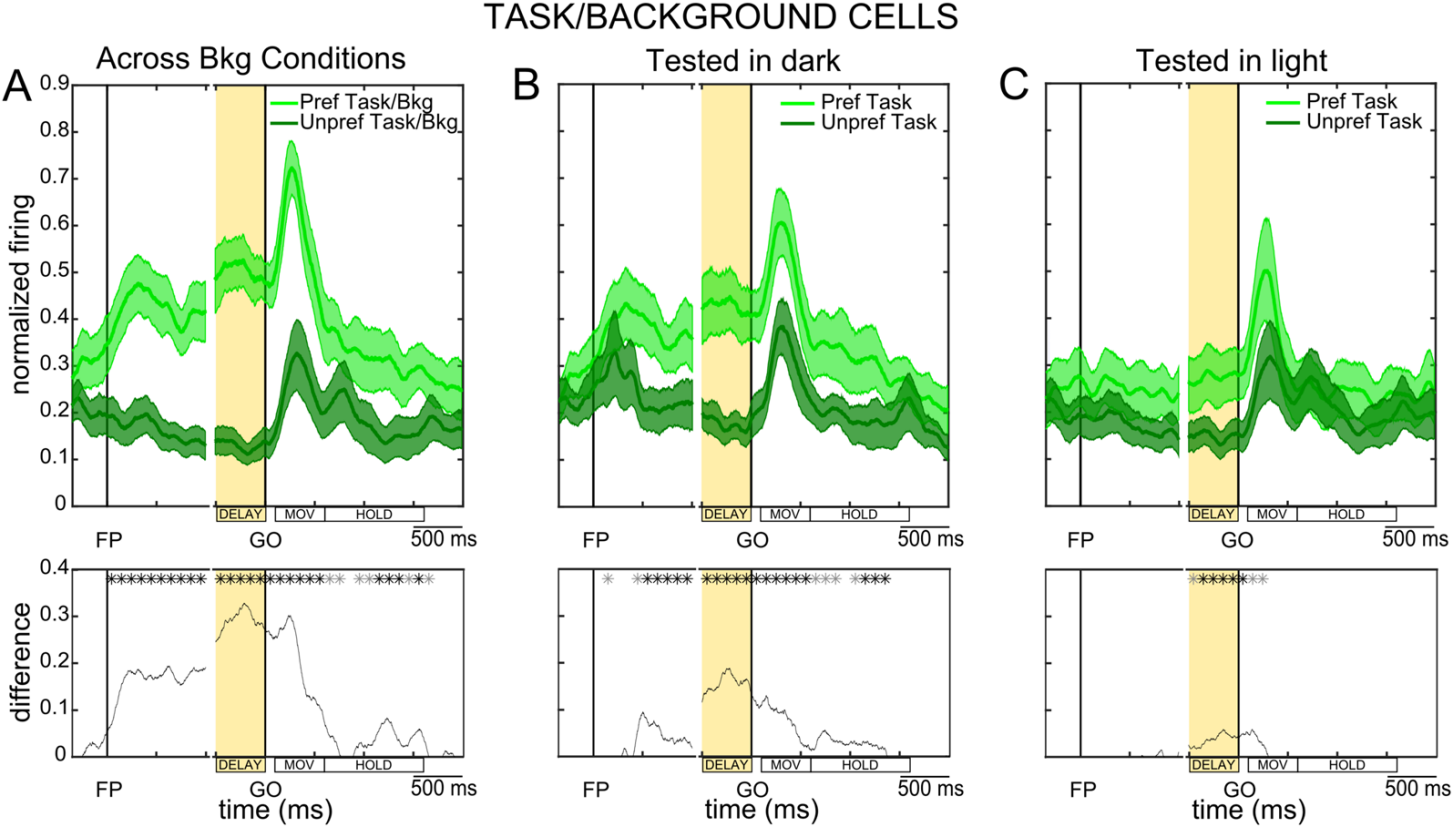
Combined selectivity for task and visual condition. (**A**) Population SDFs represent the temporal unfolding of combined task and background preference across the whole population of *Task/Background cells*, as assessed by averaging spiking activity during the preferred (in green) vs. unpreferred (in dark green) task and visual condition. (**B**) Population SDFs represent the temporal unfolding of task preference across the whole population of *Task/Background cells*, when tested with a dark background. (**C**) Population SDFs represent the temporal unfolding of task preference across the whole population of *Task/Background cells*, when tested with a light background. All conventions as in Fig. 4.

Normalized population SDFs depicted in Fig. 6A show the pattern of discharge of *Task/Background cells*, as assessed by averaging spiking activity during the preferred task and background condition vs. the unpreferred task and background condition, based on single-cell preferences. The activity during the DELAY epoch, as well as during the subsequent execution of the arm/hand movement, was reliably different in preferred vs. non-preferred task/conditions (see black line and asterisks in the lower panel of the figure). The difference was also often significant during the HOLD period, such that in Fig. 6A a clear separation between the two curves is evident along almost the entire duration of the trial. We also measured pure task-related modulations in Task/Background cells, separately when tested in the dark (Fig. 6B) and in the light (Fig. 6C); in both background conditions, activity of this group of cells was reliably different in the preferred vs. unpreferred task, although task preference was more evident when tested in the dark (Fig. 6B).

Analogously to what performed with *Task cells*, also for *Task/Background cells*, we analyzed how task preference changed along the trial by computing the TPI in sliding time windows (width: 200 ms, shift: 50 ms; see Methods) in order to gain a deeper understanding of the precise unfolding of task-related activity. Results from this analysis are reported in Fig. 5C, separately for the cells that showed a preference for the Reach-to-Press task (red line; n = 7) and for those preferring the Reach-to-Grasp task (blue line; n = 14). The maximal expression of task preference was found during DELAY for both subgroups of cells; task preference then remained substantial during the execution of arm/hand movement and during the HOLD epoch, but only for cells with a preference for the Reach-to-Grasp task (blue line), whereas it dropped more rapidly for cells with a preference for the Reach-to-Press task (red line).

Finally, and most critically, similarly to what described for *Task cells*, we tested whether the task-related modulation observed during the DELAY epoch was consistent with the preference expressed by each cell during arm/hand movement execution, this being the necessary bond for substantiating the motor-related nature of the measured pre-movement activation. Regression analyses revealed a significant correlation between TPI values measured in the two epochs (R2 = 0.57; F = 25.1; p < 0.001; Fig. 5D). Although still reliable, for *Task/Background cells* this relationship appears weaker and the regression line is less steep (slope = 0.64) with respect to that shown for *Task cells* (see Fig. 5B). Possibly, this is linked to the increased variability across time that arises from the combined encoding of different signals in this group of cells (task preference and visual feedback). In relation to the latter, it is also important to consider that, in the light condition, visual stimulation is not completely constant across the trial, because, during movement execution, the monkeys also see their own arm/hand, in addition to a stable visual environment and to the pressing/grasping target. Thus, visual stimulation will likely increase the variability of cell modulation, but also the richness of signals potentially encoded by this cell population.

### Evidence for functional clustering in V6A

**Figure 7:**
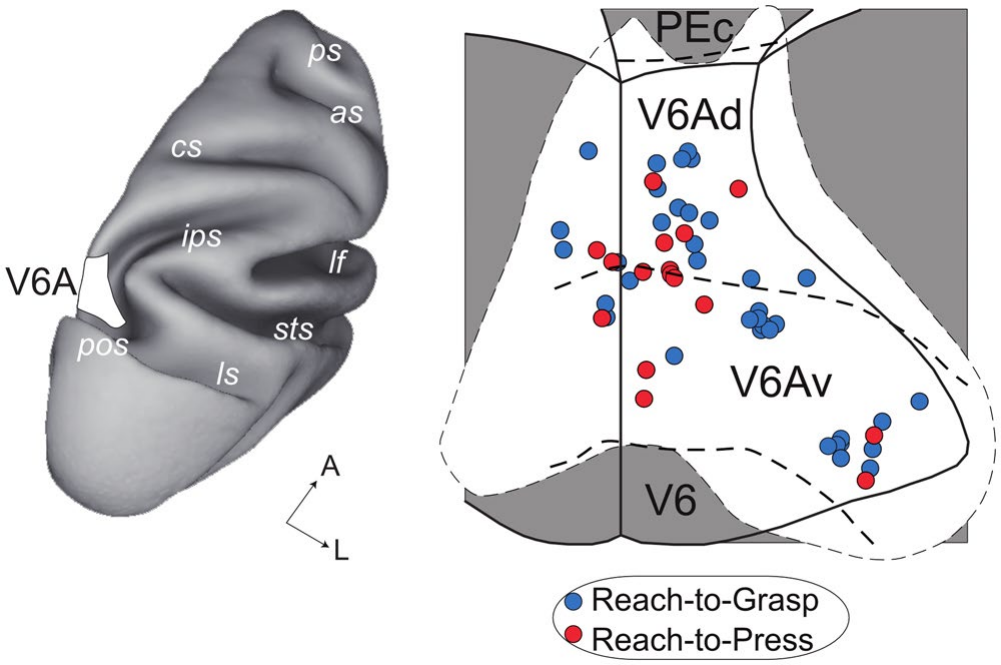
Absence of functional clustering in V6A neurons. Left, the location of area V6A in the parieto-occipital sulcus (pos) is shown in a dorso-lateral view of a hemisphere reconstructed in 3D using the Caret software (https://www.nitrc.org/projects/caret/). ls, lunate sulcus; sts, superior temporal sulcus; ips, intraparietal sulcus; cs, central sulcus; as, arcuate sulcus; ps, principal sulcus; a, anterior; l, lateral. Right, topographical representation of area V6A, with precise representation of the recording sites (location on the map) and functional properties (color coding) of the neurons (circles on the map) with reliable task-related modulations of activity during the DELAY epoch (note that *Task* cells and *Task/Background* cells are represented together, considering for both only task-related modulations). Blue and red colors indicate a preference for the Reach-to-Grasp and Reach-to-Press, respectively. Dorsal and ventral sectors of V6A (V6Ad, V6Av) are shown in the map (note that maps of each left hemisphere were flipped, so that all recording sites were projected on the maps of the right hemispheres); V6, area V6^6^; PEc, area PEc^92^. No evidence for functional clustering is present in the recorded area.

As a further step, we were interested in understanding whether V6A neurons showing specific task preferences were organized in functional clusters within the cortical extension of macaque area V6A. We therefore reconstructed the 2D-map of area V6A and reported on the map the position and task preference (as measured during the DELAY epoch) of each neuron in the relevant cell classes, i.e., *Task cells* and *Task/Background cells* (Fig. 7, superimposition of individual flattened maps of the studied animals). As evident from the inspection of Fig. 7, cells showing task-related modulations are distributed across the whole extension of V6A, and there is no functional clustering among cells showing a task preference either for the Reach-to-Press or for the Reach-to-Grasp task. To conclude, we found no evidence that separate regions within V6A do exist that are functionally dedicated to the encoding of specific arm/hand actions.

### Preparing arm/hand actions requiring or not a precise grip conformation to the object shape

**Figure 8:**
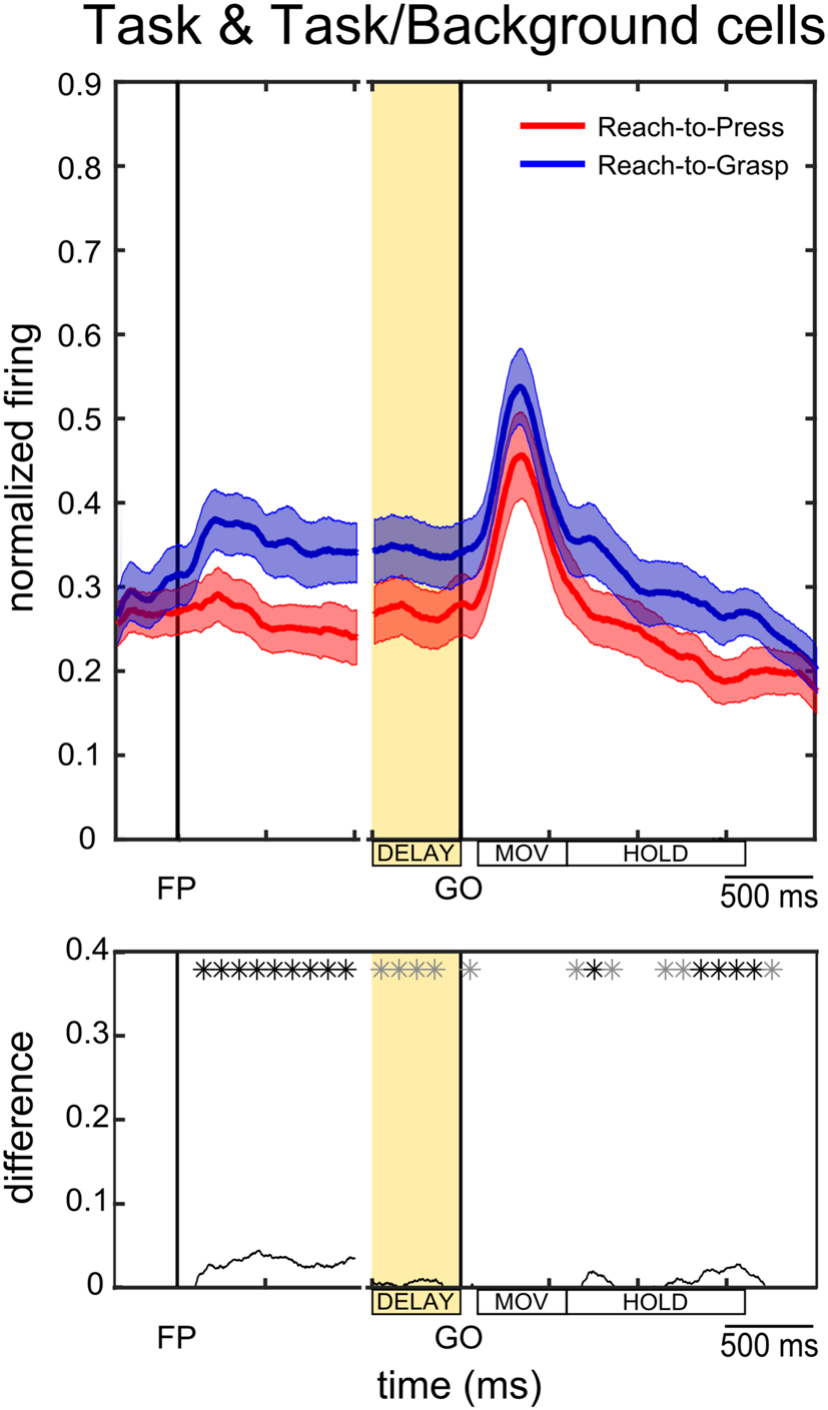
Pre-movement coding of reach-to-press and reach-to-grasp movements across task-selective cells. Population SDFs for the Reach-to-Grasp (in blue) and Reach-to-Press (in red) tasks are shown for all cells in the population which showed a task-related activity in the DELAY epoch (n = 47), i.e. both *Task cells* and *Task/Background cells*, as computed across background conditions and independently of single-cell task preference. The population of cells shows an overall preference for preparing a reach-to-grasp action, rather than a reach-to-press one. All conventions as in Fig. 4A.

In both the groups of cell described above, i.e. *Task cells* and *Task/Background cells*, we observed a prevalent selectivity for reach-to-grasp movements during the DELAY epoch. In order to assess whether this preference was reliable across the entire population of neurons showing a task effect in the pre-movement phase of the trial, we computed population activity over the course of the whole trial for *Task cells* and *Task/Background cells* together, separately for the Reach-to-Press and the Reach-to-Grasp task, independently of single cell task preference (Fig. 8). Overall, we found significantly higher population activity during the Reach-to-Grasp Task (see black line and asterisks in the lower panel of the figure), most critically during the pre-movement phase, thus confirming that delay activity in V6A has a critical role in the preparation of impending reach-to-grasp movements.

## Discussion

With the aim of testing whether V6A neurons do encode preparatory signals specifically related to action planning, we designed an experimental paradigm allowing direct comparison of neural signals emerging prior to movement execution in two instructed-delay visuomotor tasks, sharing the same spatial and temporal structure, but requiring different hand actions. More specifically, the actions required in the two tasks, a reach-to-press and a reach-to-grasp movement respectively, shared the transport component, i.e., they both involved reaching a target at the same spatial location, but critically differed in their distal component, i.e. in terms of specific hand shaping and grip formation requirements. Although both tasks involve reaching to a specific location, V6A neurons distinguish the hand actions to be performed (grasping or pressing), and V6A clearly highlights grasping both by the relative firing rates (as illustrated by stronger activity in Fig. 8), and by the total percentage of neurons showing preference for this behavior (see Results).

Moreover, data were obtained in two different visual conditions, namely when the tasks were executed in the dark and in the light, allowing us to control for any influence of visual information on pre-movement activity. In agreement with previous findings from our group^7,18,19,41^, neural activity in V6A was found to be affected by both the task at hand, i.e., the type of action required, and the presence/absence of visual feedback. Task-related signals were observed along the whole duration of each trial, notably during the instructed-delay phase and during movement execution. More relevant to the specific aim of the present study, collected findings demonstrate that, in most V6A neurons, a selective task-related preparatory activity is expressed in anticipation of action execution, which is able to effectively differentiate arm/hand movements aimed at reaching and pressing a specific target, and arm/hand movements aimed at reaching and grasping an object located at the exact same spatial location, with appropriate wrist orientation and grip formation. The activity recorded during the delay phase does not depend on factors related to visual modulation or other potential confounding factors (see Results); rather, it is strictly dependent on the specific movement to be subsequently performed. The strong consistency between task preference measured during instructed-delay and movement execution supports a tight link between the two processes, attesting to a role of the task-related delay activity in action planning. The described evidence of a differential preparatory activity in single V6A neurons provides a clear demonstration that the functional role of V6A goes beyond the online guidance of movement (as suggested by several authors^4,52,53^) to embrace action preparation and the encoding of motor intention.

In our data, during an instructed-delay epoch preceding the GO signal which allowed movement execution, neural activity in neurons encoding task preference (*Task cells* and *Task/Background cells*) clearly diverged depending on the specific action required in the given block of trials. While some neurons showed a strong preference for reach-to-grasp movements, others showed a strong preference for reach-to-press movements. In line with increasing evidence for a significant role of area V6A and, more generally, of the dorsomedial visual pathway in the encoding of grasping movements^4,19,41,42^, the majority of cells with significant task-related activity showed a preference for the Reach-to-Grasp task. Accordingly, the entire population of cells showing task effects showed higher activation while the monkey was preparing the execution of a reach-to-grasp action (see Fig. 8).

Interestingly, V6A seems to play a similar role in human and non-human primates. In fact, it has been recently proposed that area aSPOC, which is suggested to include the putative human V6A^33,35,38^, plays a significant role in the preparatory encoding of specific motor programs for reaching and grasping movements^40,54,55^.

In the present study, we collected strong evidence attesting that the functional role of the measured task-related preparatory activity is strictly related to the subsequent motor act, which clearly corroborates its motor nature. Specifically, given that each of the two visuomotor tasks was recorded both in the dark and in the light, by comparing delay activity between the two visual conditions we confirmed that task-related preparatory activity was clearly separable from visual modulations, being expressed independently of the background, notably for *Task cells*. Along the same line, an explanation of task-related preparatory activity based on the presence/absence of a visual feedback will not be substantiated for *Task/Background cells* which do show combined, yet independent, modulations. In some instances, however, the two effects might also influence one another, such that for example pre-movement task-related modulation for a given neuron could be expressed more strongly in one vs. the other of the two visual background conditions.

Another piece of evidence in strong support of a motor-related, preparatory activity in the instructed-delay phase derives from the striking correlation between task preference in the DELAY epoch (i.e., before the animal performed any action), and during the actual execution of movements, which strongly suggests that pre-movement activity was the expression of a specific motor program. In addition to being evident in *Task cells*, such correlation was observed also for *Task/Background cells* with its strength being only marginally reduced, likely, as a result of variability in the expression of different signals across time in the latter group of cells (see Results).

In spite of the strong evidence in support of a motor-related preparatory activity in V6A neurons, we are not able to fully exclude that pre-movement activity also entails an attentional component that allows spatial focusing of selective attention at the service of the action to be performed (for recent data in support of attentional effects in V6A, see^56–59^). Future experiments are needed to specifically address the effect of attention on action control in V6A.

Interestingly, the temporal dynamics of neural activity selectively encoding for reach-to-press and reach-to-grasp movements in the pre-movement phase showed some clear differences. In neurons showing a preference for Reach-to-Press, the instructed-delay activity builds up as a ramp before the GO signal (see red line in Fig. 4A). This ramp activity is similar to that described before arm movement execution in neurons within the premotor cortex^60–64^ and in other parietal regions of the brain^22,65,66^, thus supporting the view that it represents a preparation activity for the impending motor act. In neurons showing a preference for Reach-to-Grasp, we observed instead a more sustained preparatory activity, which emerged earlier and more abruptly at the beginning of the trial, often in light, but sometimes also in dark background conditions (see example in Fig. 3B). This sustained activity for grasping movement started as soon as the animal got actively engaged in the task. In the light background condition, the active engagement in the task corresponded to when the handle (which was always visible) started to be actively considered as the object to be grasped. This event likely triggers neuronal activation reflecting the encoding of the visual affordance of the object^12,17^. As reported above, a similar sustained activity after fixation was sometimes observed also in the dark background condition. In these cases, the delay activity might have been evoked by the active maintenance in working memory of the location, form and orientation of the object to be grasped, that the animal saw at the beginning of the block of trials performed in the dark, or the learned affordance of it. To remind the reader, it is also important to note that, because of the blocked design of the task protocol, secure knowledge of the specific actions required in the given trial facilitated advanced encoding of movement planning.

Importantly, apart from an initially diverging dynamics of the activity during the Reach-to-Press and Reach-to-Grasp tasks, a sustained activity of V6A neurons was always measured in the late phase of the instructed-delay, namely the DELAY epoch. This sustained activity, which was the focus of our investigation, varied according to the impeding arm/hand movement and might directly reflect motor intention, or the active maintenance of the selected motor program in the waiting period preceding the GO signal^22,67–69^.

Data collected in this study, together with previous demonstrations of grasp-related activity during movement execution^15–17^, strengthen the idea of a significant role of area V6A in the encoding of the whole prehensile action^4,32^, not limited to the online guidance of action execution, as previously suggested, but also concerning movement preparation, and notably including precise planning of hand shaping and grip configuration.

Critically, neurons in area V6A not only have a role in the online guidance of grasping actions, including by encoding wrist orientation^15^ and grip type^16,17^, but do also encode information related to the distal component of specific motor acts in a pre-movement, planning phase. Needless to say, in this perspective, the findings described here are utterly incompatible with the traditional view of a clear separation of cortical visual streams responsible for encoding reaching and grasping movements, respectively, a view that has been prominent in the field for a long time (e.g.^70^), and is still reported in several basic texts of neuroscience (e.g.^71^).

Interestingly, a very recent study using magneto-encephalography (MEG) in humans has demonstrated that both the dorsolateral (putative grasping) and the dorsomedial (putative reaching) stream play a critical role in planning of non-visually guided reaching vs. grasping actions^72^. Once again, this notion corroborates the hypothesis that grasping information is coded within both pathways, in line with multiple recent evidence collected both in human^40,54,73–75^ and monkey studies^4,19,42,76,77^.

Recent studies suggest that signals related to both reaching and grasping movements do also coexist in the anterior intraparietal area (AIP)^78–80^, an area which has long been considered the quintessential cortical substrate for neural signals allowing encoding of hand shaping for grasping execution^81–86^. Specifically, neuronal activity in AIP was recorded while monkeys were performing a grasping task in varying conditions entailing different hand grips and systematic variations of gaze and target location^78,79^. In this context, in addition to showing the expected selectivity for hand grip type and configuration, AIP neurons were found to encode spatial information relative to both gaze and target position, which are naturally linked to programming arm direction, during both movement planning and execution^78,79^.

A convincing framework for putting these recent observations together is one in which the dorsomedial and dorsolateral visual streams are considered as different, but interconnected routes for controlling the whole act of prehension, that includes both reaching and pressing/grasping movements^32^. Although these two sub-streams might subserve different functional roles, not yet fully clarified at present, they might cooperate and supplement one another in order to orchestrate an accurate planning and execution of prehensile actions^4,32^. What we report in the present paper enriches the preceding functional view of area V6A in that it extends its putative role beyond movement execution. In fact, present results provide the first evidence of a differential pre-movement activation in single V6A neurons exquisitely related to the specific motor program to be subsequently executed. The existence of such a differential preparatory activity strongly supports a role of V6A in arm/hand movement planning, notably including advanced, pre-movement encoding of wrist orientation and grip type, critical aspects that define the distal component of specific actions.

## Methods

Two male adult macaque monkeys (*Macaca fascicularis*) weighing 3.1 and 3.8 Kg, respectively, were involved in the study. During training and recording sessions, particular attention was paid to any behavioral and clinical sign of pain or distress. The study was performed in accordance with the guidelines of the EU Directives (86/609/EEC; 2010/63/EU) and the Italian national law (D.L. 116-92, D.L. 26-2014) on the use of animals in scientific research. Protocols were approved by the Animal-Welfare Body of the University of Bologna.

### Behavioral paradigm

We trained the animals to perform two instructed-delay visuomotor tasks, sharing the same temporal structure and procedure, but requiring different actions to be performed, namely a *Reach-to-Grasp task* (Fig. 1A), and a Reach-to-Press *task* (Fig. 1B). In both cases, the monkeys performed coordinated arm/hand reaching movements with the limb controlateral to the recording side, with the head restrained and while maintaining steady fixation on a light-emitting diode (LED) placed straight-ahead at eye level (fixation LED). At the end of the reaching movement, the animals grasped a handle in the Reach-to-Grasp task, whereas they pressed a button at the very same spatial location in the Reach-to-Press task. For both animals, the two tasks were performed in the dark (Fig. 1, top) and in the light (Fig. 1, bottom), thus collecting data in different background conditions. The two visuomotor tasks were executed in separate block of trials, with the order of execution being counter-balanced across experimental sessions. Moreover, for both tasks, the dark and light background conditions were performed in separate blocks of trials arranged in a pseudorandom series.

#### Reach-to-Grasp task

In the *Reach-to-Grasp task* (Fig. 1A), the animals were trained to reach and grasp a horizontal handle (length: 4 cm) placed on a frontal panel, at a distance of 15 cm from their eyes; the horizontal handle was positioned just below the fixation LED. During the execution of the task in the dark (Fig. 1A, upper panels), due to the feebleness of the LED light, the monkey was not able to see either the handle or its own hand moving towards it (the handle was visible to the animal only before the beginning of the given block of trials for this task). The execution of the reach-to-grasp movement in that condition was therefore aimed to the well-learned and constant handle position, and was based uniquely on memory guidance. Instead, in the light background condition (Fig. 1A, lower panels), the handle, the reach-to-grasp action, and the final hand position were constantly visible, allowing for continuous visual feedback.

In both background conditions, each trial sequence began when the monkey pressed a button (home-button; 2.5 cm in diameter), placed outside the animal’s field of view, 5 cm in front of the chest, on the animal’s midsagittal line. After button press, the animal awaited instructions while still being free to move the eyes (FREE; Fig. 1A). After 0.5–1 s, a green fixation LED lit up and the monkey was required to fixate it (FIX; Fig. 1A) and to maintain fixation thereafter, for the entire duration of the trial sequence. Breaking of fixation and premature button release would have interrupted the trial. Subsequently, after a variable delay period (1–2.5 s; INSTRUCTED-DELAY; Fig. 1A), the LED color changed from green to red (GO; Fig. 1A), which constituted the GO signal instructing the monkey to perform the required reach-to-grasp movement. Specifically, following the GO signal, the monkey released the home-button and performed an arm/hand movement to reach and grasp the horizontal handle (MOVEMENT EXECUTION; Fig. 1A), pull it and maintain it pulled (HOLD; Fig. 1A) until the red LED switched off again. The LED switch-off instructed the monkey to release the handle and to press again the home-button in order to obtain a liquid reward in turn for correct performance and to then start a new trial sequence.

#### Reach-to-Press task

In the *Reach-to-Press task* (Fig. 1B), the animals were trained to reach and press a small button (4 mm-diameter; 1.6° of visual angle) corresponding to the fixation LED, which was mounted on a micro-switch embedded in a frontal panel, placed at a distance of 15 cm from their eyes. In line with what was described for the Reach-to-Grasp task, also the Reach-to-Press task was performed in two different background conditions. Again, in the dark (Fig. 1B, upper panels) the monkey was not able to see its own arm/hand moving towards the target button, i.e. the fixation LED, while a constant visual feedback was instead allowed when the task was executed in light (Fig. 1B, lower panels).

It is worth noting that the trial sequence was identical to the one described above for the Reach-to-Grasp task. The only element differentiating the two tasks was the type of coordinated arm/hand movement required. In the case of the Reach-to-Press task, the GO signal instructed the monkey to reach and press the target for the entire HOLD period, until the target itself switched off. In this task, the monkey did not shape the hand to grasp any object.

In both tasks, the correct performance of the monkeys was detected online by press/release of micro-switches (monopolar micro-switches, RS Components, UK) mounted under the home-button, the fixation LED and the handle.

### Surgical procedures and electrophysiological recordings

Following an initial phase in which the monkeys got used to sit in a primate chair and to interact with the experimenters, a head-restraint system and a recording chamber were surgically implanted in conditions of asepsis and under general anesthesia (sodium thiopenthal, 8 mg/kg*h, i.v.). A full program of postoperative analgesia (ketorolac trometazyn, 1 mg/kg, *i.m*., immediately after surgery, and 1.6 mg/kg, *i.m*., on the following days) and antibiotic care [Ritardomicina® (benzathine benzylpenicillin + dihydrostreptomycin + streptomycin) 1–1.5 ml/10 kg every 5–6 days] was applied after surgery. After full recovery, the monkeys were trained on the behavioral tasks. A more detailed description of surgical procedures is reported elsewhere^48^.

Single cell activity was extracellularly recorded from the posterior parietal area V6A^6^ (Fig. 7). We performed single microelectrode penetrations using glass-coated metal microelectrodes with a tip impedance of 0.8–2 MΩ at 1 KHz. The electrode signals were amplified (at a gain of 10,000) and filtered (bandpass between 0.5 and 5 KHz). Action potentials were isolated using a window discriminator (Bak Electronics). While the experimenter was carrying out the isolation of single neurons for recordings, monkeys were typically at rest and allowed to freely move both the eyes and limbs. This procedure allowed us to avoid the introduction of any sampling bias in the isolation of neurons. Recording procedures used for one monkey were similar to those previously reported by Galletti and colleagues^48^, with spike times being sampled at 1 KHz. Recording procedures for the second monkey were slightly different, with spikes being sampled at 100 KHz, and are described in details in^87^. During experimental sessions, eye movements were simultaneously recorded using an infrared oculometer (Dr Bouis, Karlsruhe, Germany) and sampled at 100 Hz for the first monkey and 500 Hz for the second one. Throughout the execution of the task, eye position was monitored by an electronic window (5° x 5°) centered on the fixation target. Behavioral events were recorded with a 1ms-resolution.

In order to perform accurate reconstructions of microelectrode penetrations, electrolytic lesions (40–50 μA cathodal current for 30 s) were made during the final days of recordings at different depths along single penetrations and at different coordinates within the recording chamber. After completion of data collection, the animals were anaesthetized with ketamine idrochloride (15 mg/ kg i.m.) followed by an i.v. lethal injection of sodium thiopental and perfused through the left cardiac ventricle with 0.9% solution of sodium chloride followed by 4% paraformaldehyde in 0.1 M phosphate buffer (pH 7.4) and by 5% glycerol in the same buffer. The histological reconstruction of recording sites was then performed following the procedure described in previous papers from our laboratory^11^.

### Data Analysis

Recorded neurons were included in the analysis if at least 7 correct repetitions (the actual number of repetitions was on average 14.1 per condition, across the whole sample of cells) for each experimental condition (for a total of four, including two tasks and two background conditions; see above) were collected^49^ and if the quality of recordings was high and stable throughout the recordings^14^.

Neural activity of V6A neurons was analyzed in terms of average firing rate in two time epochs: during DELAY, which was selected as the last 500 ms before the GO signal, thus corresponding to the last part of the instructed-delay phase, and during MOV, from −200 ms before movement onset to movement end.

In order to statistically estimate task-related and background-related activity, a repeated-measure sliding-window ANOVA, comprising the factors *Task* (Grasping vs. Reaching) and *Background* (dark vs. light), was applied at multiple time points along each trial. Specifically, we applied the ANOVA to average spiking activity in 200 ms windows, and this test was repeated in time steps of 50 ms^51^. The percentage of cells in the population showing a significant main effect of Task or a significant main effect of Background (p < 0.05) was calculated for each time window.

To focus on how task preference and background preference were expressed in the neuronal population during the critical pre-movement phase of arm/hand actions, we then applied the same repeated-measure ANOVA to average spiking activity in the DELAY epoch. As a result, groups of neurons selectively showing reliable task preferences (*Task cells*), background preferences (*Background cells*), or both (*Task/Background cells*), were identified, and their activity was then analyzed in greater detail.

To illustrate the time course of task-related modulations in the above mentioned groups of neurons, population responses were computed as average spike density functions (SDFs). A SDF was calculated (Gaussian kernel, half-width: 80 ms) for each neuron included in the analysis, by averaging across all trials for each tested condition. Peak discharge rate of the neuron was calculated and used to normalize the SDF. Normalized SDFs where then averaged to derive population responses^18^. To assess consistency of the results across cells in the population, we applied a subsampling method^88,89^ for bootstrapping data. Specifically, random subsamples of cells were extracted (without replacement) 10,000 times from the original population, with the size of the subsample corresponding to two thirds of the size of the population of interest (see Results). Confidence intervals (CI) were estimated as the range that delimited 95% of the computed SDF values from the 10,000 newly generated subsets^90^. In addition, in all cases, when analyzing the time course of the effects for the population of interest, we performed non-parametric statistical comparisons (two-tailed paired Wilcoxon tests comparing experimental conditions, performed on the activity measured in consecutive 100 ms time windows along the whole duration of the trial) beginning after the presentation of the fixation LED (epoch FIX), i.e. the signal requiring active engagement of the monkey at the beginning of each trial.

Task-related modulations were also quantified by calculating an *ad-hoc* index^91^, the Task Preference Index (TPI), which was calculated as:

$$\frac{({\boldsymbol{Press}}\,-\,{\boldsymbol{Grasp}})}{({\boldsymbol{Press}}\,+\,{\boldsymbol{Grasp}})},$$

where *Press* is the mean activity of the neuron measured during the considered time window (see below) in the Reach-to-Press task, averaged across background conditions, and *Grasp* is the mean activity of the neuron measured during the considered time window in the Reach-to-Grasp task, again averaged across background conditions. Note that positive TPI values correspond to a preference for the Reach-to-Press task, while negative values correspond to a preference for the Reach-to-Grasp task. The described index was calculated both in the two epochs of interested mentioned above, namely the DELAY and MOV epoch, and in overlapping time windows (width: 200 ms; shift: 50 ms) spanning the entire duration of each trial, with the aim of performing fine grained temporal analyses.

To assess consistency in task preference across critical time windows along the trial, we plotted the TPI calculated for single-cell activity measured during the DELAY epoch against the TPI calculated for single-cell activity measured during the MOV epoch, and performed a linear regression analysis, employing ordinary least square as the estimation method. Note that, given a statistically significant regression line (p < 0.01), a slope value close to 1 indicates highly consistent task preference across critical epochs at the single-cell level. Conversely, a slope value close to zero would indicate non-consistent results across the two epochs.

### Data availability

The datasets generated during and/or analyzed during the current study are available from the corresponding author on reasonable request.
